# Burden of chronic kidney disease among children and adolescents in the South-East Asia and Western Pacific regions, 1990 to 2021

**DOI:** 10.3389/ijph.2026.1609313

**Published:** 2026-09-16

**Authors:** Yang Yang, Yujie Xu, Xiaoran Xue, Mingyi Zhao, Qingnan He

**Affiliations:** Department of Pediatrics, The Third Xiangya Hospital, Central South University, Changsha, China

**Keywords:** chronic kidney disease, disability-adjusted life-years, GBD 2021, global burden of diseases, socio-demographic index

## Abstract

**Objectives:**

There is little comprehensive or updated evidence on the burden of CKD among children and adolescents in Asia-Pacific. We aimed to report the CKD burden among people under 20 years from 1990 to 2021 using data from the Global Burden of Diseases, Injuries, and Risk Factors Study 2021 (GBD).

**Methods:**

We conducted a retrospective descriptive analysis using GBD 2021 data for individuals aged <20 years in the South-East Asia and Western Pacific regions. We assessed age-standardized rates (ASRs) of CKD prevalence, incidence, deaths, and disability-adjusted life years (DALYs) from 1990 to 2021, and examined their associations with the Socio-demographic Index (SDI).

**Results:**

In 2021, the ASR of CKD incidence was 26.72 per 100,000 in South-East Asia and 20.92 per 100,000 in the Western Pacific. From 1990 to 2021, overall ASRs of CKD incidence, deaths, and DALYs declined in both regions (AAPC for DALYs: −2.43 in South-East Asia and −2.87 in Western Pacific). Children aged <5 years had the highest incidence rates (53.97 and 49.29 per 100,000), whereas adolescents aged 15–19 years had the highest burden in terms of prevalence (2,630.63 and 1,445.9 per 100,000), deaths (1.03 and 0.91 per 100,000), and DALYs (93.33 and 76.65 per 100,000). Males experienced higher death and DALY rates than females in both regions (death rate: 0.79 and 0.51 vs. 0.55 and 0.42 per 100,000; DALY rate: 73.25 and 44.64 vs. 52.65 and 38.69 per 100,000). CKD burden declined more markedly in high-SDI countries. However, several Pacific Island countries, despite being classified as middle- or high-SDI, exhibited disproportionately high CKD burden due to geographic and healthcare access challenges.

**Conclusion:**

CKD burden in children and adolescents in Asia-Pacific decreased from 1990 to 2021. Gender- and age-specific interventions and early treatment of CKD-related complications may help improve patient care, especially in regions with lower SDI and Pacific Island countries.

## Introduction

In recent years, the prevalence of chronic kidney disease (CKD) among children and adolescents has been steadily increasing, emerging as a growing challenge in global public health [1]. Although this age group accounts for a relatively small proportion of the overall CKD population, once CKD occurs, it tends to progress rapidly with insidious early symptoms, often being diagnosed at an advanced stage [2]. This has a profound impact on physical development, mental health, and quality of life in adulthood, while also imposing a substantial economic and caregiving burden on families and society [3]. According to estimates by the World Health Organization (WHO), approximately 800 million people worldwide are affected by CKD [4]. However, most existing epidemiological studies have focused on adult populations, and the burden of CKD among children and adolescents, especially in low- and middle-income countries in the South-East Asia and Western Pacific regions, remains insufficiently characterized.

Recent studies have shown a continued rise in the prevalence of CKD in children and adolescents globally [1], with diverse and complex etiologies, including congenital anomalies of the kidney and urinary tract, as well as secondary causes such as infections, autoimmune disorders, and metabolic diseases [5, 6]. Across different countries and regions, marked heterogeneity exists in the epidemiological patterns, clinical pathways, and outcomes of pediatric CKD, due to variations in population structure, healthcare resource allocation, and access to medical services [7]. In resource-limited settings, the lack of systematic screening and early intervention often leads to delayed diagnosis and progression to end-stage renal disease (ESRD) [8, 9]. Although kidney replacement therapy (KRT), including dialysis and transplantation, provides essential treatment for ESRD, its applicability, accessibility, and long-term outcomes in pediatric populations remain constrained by numerous challenges [10].

The South-East Asia and Western Pacific regions are among the most densely populated and socioeconomically diverse areas in the world. Considerable disparities in healthcare system capacity, disease surveillance, and policy responsiveness among countries further complicate the epidemiological profile of pediatric CKD in this region, making disease prevention and management particularly difficult [11]. Although some national or hospital-based data reports exist in this region—for example, on pediatric CKD cases in Thailand [12], Singapore [13], and certain areas of China [14]—there is a general lack of systematic studies that span multiple countries, cover broad age groups, and track long-term trends. Most of the existing studies are based on local samples, with limitations such as insufficient representativeness, inconsistent indicators, and a lack of time series data, making them inadequate for robust policy support. In addition, the incidence of pediatric CKD in this region is influenced by a series of unique risk factors, such as chronic malnutrition, contaminated drinking water, high burdens of infectious diseases, and insufficient coverage of primary healthcare services [11]. These region-specific factors may contribute to kidney damage through multiple pathways, including environmental toxin exposure, recurrent infections, and impaired growth and development in early life. Moreover, the distribution and intensity of these risk factors vary substantially across countries and between urban and rural areas, reflecting underlying socioeconomic and environmental inequalities. To address this gap, the present study utilizes data from the Global Burden of Disease (GBD) study to systematically evaluate the burden of CKD among individuals aged 0–19 years in the South-East Asia and Western Pacific regions from 1990 to 2021. We examine trends in incidence, mortality, and disability-adjusted life years (DALYs), as well as geographical and sex-specific disparities, and explore the role of sociodemographic factors in shaping the disease burden. By identifying gaps in current prevention and control systems, this study aims to inform regional health policy development, promote early intervention strategies, and support the optimization of pediatric kidney care, thereby advancing awareness and attention to CKD in children and adolescents across and beyond these regions.

## Methods

### Overview

GBD 2021 provides the epidemiology data and burden estimation of 371 diseases and injuries for 204 countries and territories [15]. The disease indicators include prevalence, incidence, death, years lived with disability (YLDs), years of life lost (YLLs), and disability-adjusted life years (DALYs). GBD synthesizes the raw data extracted from censuses, surveys, demographic data, and other health-related data sources, and uses the Cause of Death Ensemble model, Spatiotemporal Gaussian process regression, and a Bayesian meta-regression modeling tool to generate consistent disease burden estimates [16]. The detailed data sources and methods used in GBD 2021 have been reported elsewhere [15].

We conducted a secondary analysis using CKD 2021 data on prevalence, incidence, death, and DALYs for children and adolescents younger than 20 years in 42 countries and territories of the WHO South-East Asia and Western Pacific regions (Supplementary Table S1). All estimates were downloaded from the Global Burden of Disease Results Tool (https://vizhub.healthdata.org/gbd-results/). GBD employs several standardized modeling approaches to generate disease burden estimates. The CODEm combines multiple models to estimate cause-specific mortality, selecting the best based on predictive performance. DisMod-MR is a Bayesian meta-regression tool that integrates available data on incidence, prevalence, remission, and mortality, ensuring consistency across epidemiological parameters. ST-GPR smooths estimates over space and time, improving accuracy in data-sparse settings [17]. All estimates include 95% uncertainty intervals reflecting data and model uncertainty. The hierarchical modeling approach allows estimation even when data are limited, with no exclusions due to data quality or availability.

### Data and definition

The causes of CKD covered by GBD include glomerulonephritis, hypertension, type 1 diabetes mellitus (DM), type 2 DM, and unknown causes. However, there is no data on CKD caused by type 2 DM and hypertension in children under 15 years old in GBD. GBD 2021 generates estimates for CKD burden by gender, age, location, and year. To provide a general insight into the CKD burden, we retrieved the estimates of prevalence, incidence, death, and DALYs of CKD in these two regions. Based on the estimates provided by GBD, we used linear regression to calculate age-specific rates and their average annual percentage changes (AAPC) using log ratios as the dependent variable and each year as the independent variable [1] to assess the trend over time. We also performed analyses by gender (female and male) and age (0–4 years, 5–9 years, 10–14 years, and 15–19 years) at regional and national levels.

### Sociodemographic index

SDI is a new developmental categorical indicator proposed by the Institute for Health Metrics and Evaluation (IHME). It consists of the total fertility rate up to age 25 (TFU 25), lagged disposable income *per capita* (LDI), and average educational attainment for ages 15 and older (EDU 15+), and is closely related to population health outcomes and social development status. A value of one indicates that the TFU is up to 25, the LDI is the highest, and the EDU is the highest, implying that the region has the highest level of theoretical development related to health outcomes. The opposite is true for an SDI value of 0. The SDI categorizes countries and regions into five categories: high SDI, high-middle SDI, middle SDI, low-middle SDI, and low SDI levels [15]. We explored the association of the rate of CKD death and DALYs with SDI using linear regression analyses.

### Statistical analysis

All statistical analyses, including regression analysis and the calculation of AAPC, were performed using R software (version 4.4.0).

## Results

### Burden of CKD in the Asia-Pacific region among individuals aged 1–19 years

In the South-East Asia region, the prevalence rate of CKD continued to increase from 785.2/100000 in 1990 to 920.36/100000 in 2019 [AAPC 0.52 (95% CI: 0.49–0.56)] (Table 1). However, the prevalence of CKD in the Western Pacific region showed a continuous decreasing trend from 559.62/100000 in 1990 to 475.41/100000 in 2021 [AAPC -0.52 (95% CI: −0.69 to −0.35)]. The incidence, death, and DALYs rates decreased both in these two regions from 1990 to 2021. Overall, the disease burden of CKD in the South-East Asia region was higher than that in the Asia Pacific region.

**TABLE 1:** The rate of chronic kidney disease prevalence, incidence, death, and disability-adjusted life-years (DALYs) in children and adolescents from 1990 to 2021 (South-East Asia region and Western Pacific region. 1990–2021).

​	​	1990 (95% CI)	2021 (95% CI)	Total percentage change in rate, 1990–2021	AAPC in rate, 1990–2021
South-East Asia region					
​	Prevalence	785.2 (649.37–948.77)	920.36 (752.28–1,121.97)	0.17 (0.15–0.2)	0.52 (0.49–0.56)
​	Incidence	27.93 (23.84–32.54)	26.72 (22.43–31.76)	−0.04 (−0.11 to 0.02)	−0.15 (−0.2 to −0.09)
​	Death	1.5 (0.85–1.82)	0.67 (0.54–0.79)	−0.55 (−0.65 to −0.2)	−2.56 (−2.92 to −2.19)
​	DALYs	135.65 (79.69–163.25)	63.33 (51.75–72.89)	−0.53 (−0.63 to −0.21)	−2.43 (−2.76 to −2.1)
Western pacific region					
​	Prevalence	559.62 (468.94–673.68)	475.41 (392.72–581.47)	−0.15 (−0.18 to −0.13)	−0.52 (−0.69 to −0.35)
​	Incidence	24.34 (20.82–28.53)	20.92 (17.64–24.94)	−0.14 (−0.19 to −0.09)	−0.46 (−0.55 to −0.38)
​	Death	1.15 (0.94–1.28)	0.47 (0.4–0.52)	−0.59 (−0.65 to −0.53)	−2.94 (−3.17 to −2.7)
​	DALYs	100.63 (83.27–112.43)	41.84 (36.63–46.91)	−0.58 (−0.64 to −0.52)	−2.87 (−3.12 to −2.63)

### CKD burden by sex and age group

In 2021, males showed a higher prevalence and incidence rate than females in the South-East Asia, while the situation in the Asia Pacific region is the opposite (Table 2). From 1990 to 2021, the CKD prevalence increased in both males [AAPC 0.52 (95%CI: 0.43–0.61)] and females [AAPC 0.56 (95% CI: 0.48–0.63)] in the South-East Aisa region, while decreased in both males [AAPC -0.35 (95% CI: −0.59 to −0.11)] and females [AAPC -0.43 (95% CI: −0.68 to −0.19)] in the Western Pacific region. The incidence rate among males in the South-East Asia region increased slightly [AAPC 0.1 (95% CI: 0.01–0.2)]. CKD death and DALYs rates declined in both two regions.

**TABLE 2:** The prevalence, incidence, death, and disability-adjusted life-years (DALYs) of chronic kidney disease by sex and age groups (South-East Asia region and Western Pacific region. 1990 and 2021).

​	​	South-East Asia region			Western pacific region		
​	​	1990 (95% CI)	2021 (95% CI)	AAPC in rate, 1990–2021	1990 (95% CI)	2021 (95% CI)	AAPC in rate, 1990–2021
Prevalence							
​	Sex	​	​	​	​	​	​
​	Female	763.8 (919.88–634.12)	905.89 (1,106.36–740.57)	0.56 (0.48–0.63)	594.39 (713.98–498.19)	498 (608.92–411.87)	−0.43 (−0.68 to −0.19)
​	Male	805.27 (976.56–666.49)	933.79 (1,137.83–767.04)	0.52 (0.43–0.61)	527.22 (635.27–441.83)	455.29 (557.83–375.67)	−0.35 (−0.59 to −0.11)
​	Age	​	​	​	​	​	​
​	0–4	43.4 (49.71–37.89)	35.04 (42.31–29.36)	−0.83 (−0.91 to −0.75)	43.65 (48.17–38.81)	29.37 (34.47–24.6)	−1.47 (−1.58 to −1.36)
​	5–9	188.63 (231.4–156.62)	160.82 (205.52–128.6)	−0.58 (−0.7 to −0.46)	164.13 (191.58–142.49)	113.18 (144.1–90.95)	−1.21 (−1.24 to −1.18)
​	10–14	716.21 (890.27–560.77)	678.29 (855.58–520.11)	−0.17 (−0.21 to −0.13)	487.65 (601.08–397.05)	415.53 (528.5–318.08)	−0.54 (−0.56 to −0.51)
​	15–19	2,635.42 (3,332.48–2079.63)	2,630.63 (3,344.26–2062.29)	0.03 (−0.04–0.1)	1,438.83 (1809.14–1,132.86)	1,445.9 (1862.12–1,096.94)	0.03 (−0.06–0.13)
Incidence							
​	Sex	​	​	​	​	​	​
​	Female	30.06 (35.27–25.65)	26.47 (31.7–22.24)	−0.44 (−0.49 to −0.39)	26.2 (30.87–22.32)	22.33 (26.77–18.77)	−0.18 (−0.48 to 0.13)
​	Male	25.92 (30.23–22.1)	26.94 (31.94–22.61)	0.1 (0.01–0.2)	22.6 (26.34–19.22)	19.67 (23.39–16.43)	−0.13 (−0.4 to 0.15)
​	Age	​	​	​	​	​	​
​	0–4	60.26 (72.26–50.46)	53.97 (64.36–45.06)	−0.42 (−0.51 to −0.33)	67.32 (79.5–56)	49.29 (58.88–40.52)	−0.81 (−0.98 to −0.63)
​	5–9	13.14 (21.15–6.72)	13.67 (21.9–7.21)	0.1 (0.01–0.2)	9.19 (16.33–4.24)	10.38 (17.32–5.32)	0.52 (0.47–0.57)
​	10–14	14.59 (24.38–7.34)	17.89 (27.54–10.08)	0.64 (0.6–0.68)	8.96 (16.14–3.89)	11.84 (19.38–6.36)	1.01 (0.83–1.2)
​	15–19	19.09 (30.5–9.23)	24.19 (35.38–13.64)	0.74 (0.68–0.81)	11.11 (19.35–4.71)	14.64 (23.12–7.62)	1.06 (0.95–1.17)
Death							
​	Sex	​	​	​	​	​	​
​	Female	1.08 (1.32–0.84)	0.55 (0.67–0.46)	−2.23 (−2.36 to −2.09)	1.08 (1.21–0.94)	0.42 (0.48–0.38)	−3.11 (−3.19 to −3.03)
​	Male	1.89 (2.45–0.72)	0.79 (0.97–0.56)	−2.79 (−2.86 to −2.72)	1.22 (1.39–0.85)	0.51 (0.57–0.42)	−2.86 (−2.92 to −2.79)
​	Age	​	​	​	​	​	​
​	0–4	2.63 (3.41–1.17)	0.75 (0.97–0.52)	−4.01 (−4.16 to −3.85)	1.65 (1.92–1.25)	0.38 (0.45–0.31)	−4.8 (−5.13 to −4.46)
​	5–9	1.11 (1.5–0.42)	0.44 (0.58–0.23)	−2.9 (−3.24 to −2.56)	0.75 (0.85–0.59)	0.28 (0.33–0.24)	−3.03 (−3.2 to −2.86)
​	10–14	0.74 (0.86–0.53)	0.47 (0.56–0.39)	−1.34 (−1.55 to −1.13)	0.68 (0.77–0.56)	0.36 (0.41–0.3)	−1.94 (−2.13 to −1.75)
​	15–19	1.37 (1.62–1.16)	1.03 (1.22–0.88)	−0.97 (−1.14 to −0.81)	1.43 (1.64–1.22)	0.91 (1.02–0.76)	−1.63 (−1.9 to −1.36)
DALYs							
​	Sex	​	​	​	​	​	​
​	Female	98.79 (119.61–77.78)	52.65 (62.65–45.02)	−2.09 (−2.21 to −1.96)	95.42 (107.65–83.64)	38.69 (43.73–34.12)	−3 (−3.06 to −2.94)
​	Male	170.21 (219.45–71.99)	73.25 (88.48–52.28)	−2.74 (−2.81 to −2.68)	105.5 (120.62–75.03)	44.64 (50.68–38.91)	−2.81 (−2.88 to −2.75)
​	Age	​	​	​	​	​	​
​	0–4	236.11 (304.59–106.68)	69.77 (88.93–49.42)	−3.9 (−4.08 to −3.73)	148.63 (172.28–112.62)	35.28 (41.91–29.32)	−4.65 (−5.02 to −4.27)
​	5–9	100.41 (132.66–42.18)	43.94 (55.7–25.86)	−2.66 (−2.96 to −2.36)	67.41 (75.98–55.01)	27.14 (30.96–23.54)	−2.85 (−3 to −2.7)
​	10–14	69.86 (80.53–51.78)	45.74 (53.61–39.13)	−1.23 (−1.41 to −1.06)	61.27 (70.24–50.83)	32.85 (38.25–28.24)	−1.93 (−2.09 to −1.77)
​	15–19	122.02 (143.36–104.62)	93.33 (111.13–80.66)	−0.94 (−1.08 to −0.8)	118.91 (135.79–99.62)	76.65 (86.99–66.62)	−1.61 (−1.84 to −1.37)

The highest incidence rate of CKD was among children under 5 years old, but the incidence rate in this population decreased from 1990 to 2021 (AAPC -0.42 in the South-East Asia region and −0.81 in the Asia Pacific region). However, other age groups all were on an upward trend. The most significant increase was found in 15–19 years age group (AAPC 0.74 in the South-East Asia region and 1.06 in the Asia Pacific region). CKD death and DALYs rates declined in all age groups.

### CKD burden by region and country

Even though the Western Pacific region showed lower prevalence, incidence, death, and DALYs rates of CKD than the South-East region (Figures 1, 2), the Pacific Island countries had a relatively higher CKD incidence and death among all countries/regions. The highest incidence rates were found in Palau, Micronesia, and Guam, and the highest death rates were in Niue, Tokelau, and Fiji. The largest increase of incidence was in Palau with a AAPC of 1.16 and the largest increase of death was in Niue with a AAPC of 0.63 (Supplementary Tables S2–5). This indicated the differences between the regional and specific country situations. Japan and the Republic of Korea had the lowest CKD disease burden, with the lowest incidence and death rates.

**FIGURE 1:**
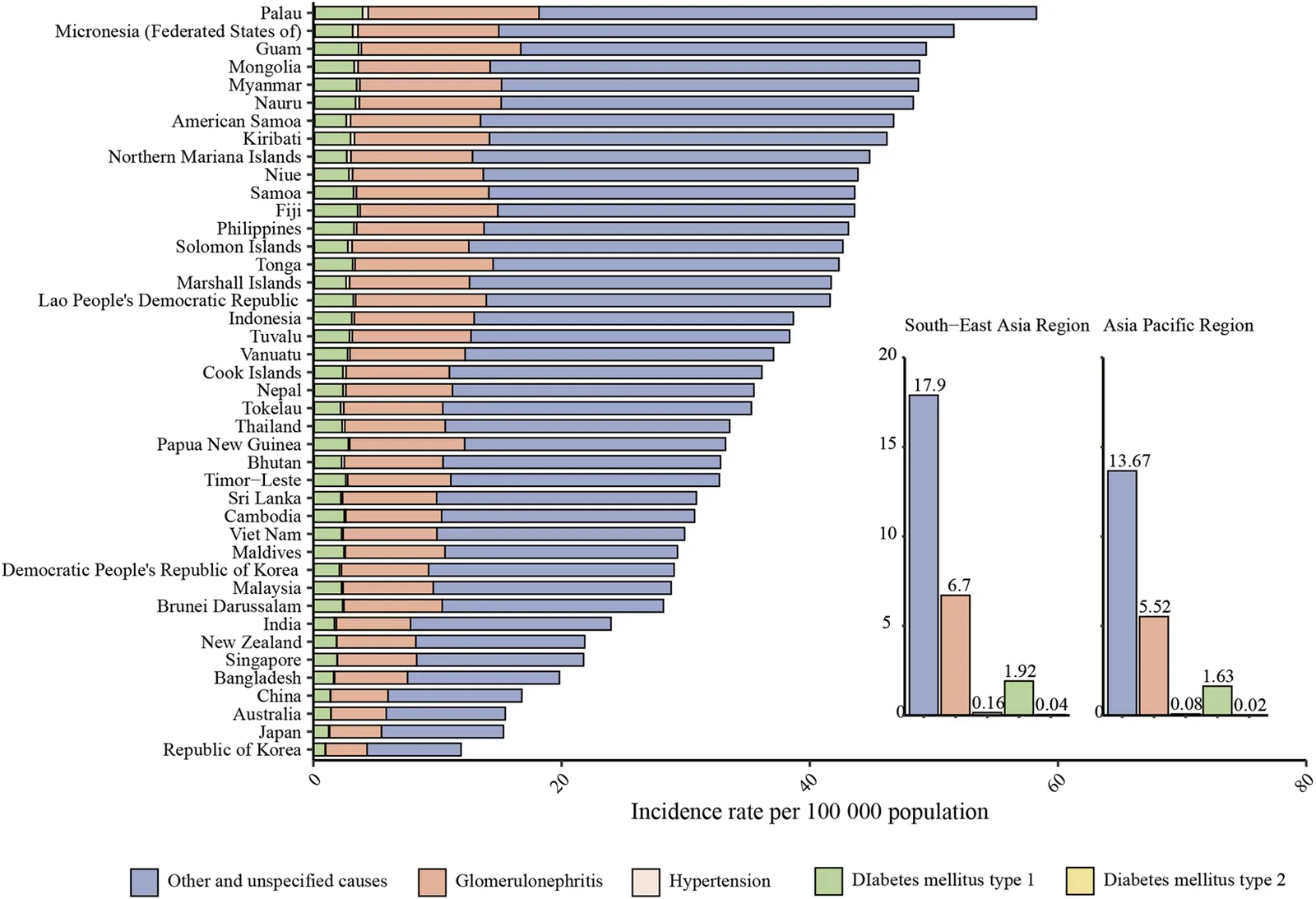
Chronic kidney disease incidence rate in children and adolescents in Asia-Pacific by country in 2021 (All Asia-Pacific countries and regions. 2021).

**FIGURE 2:**
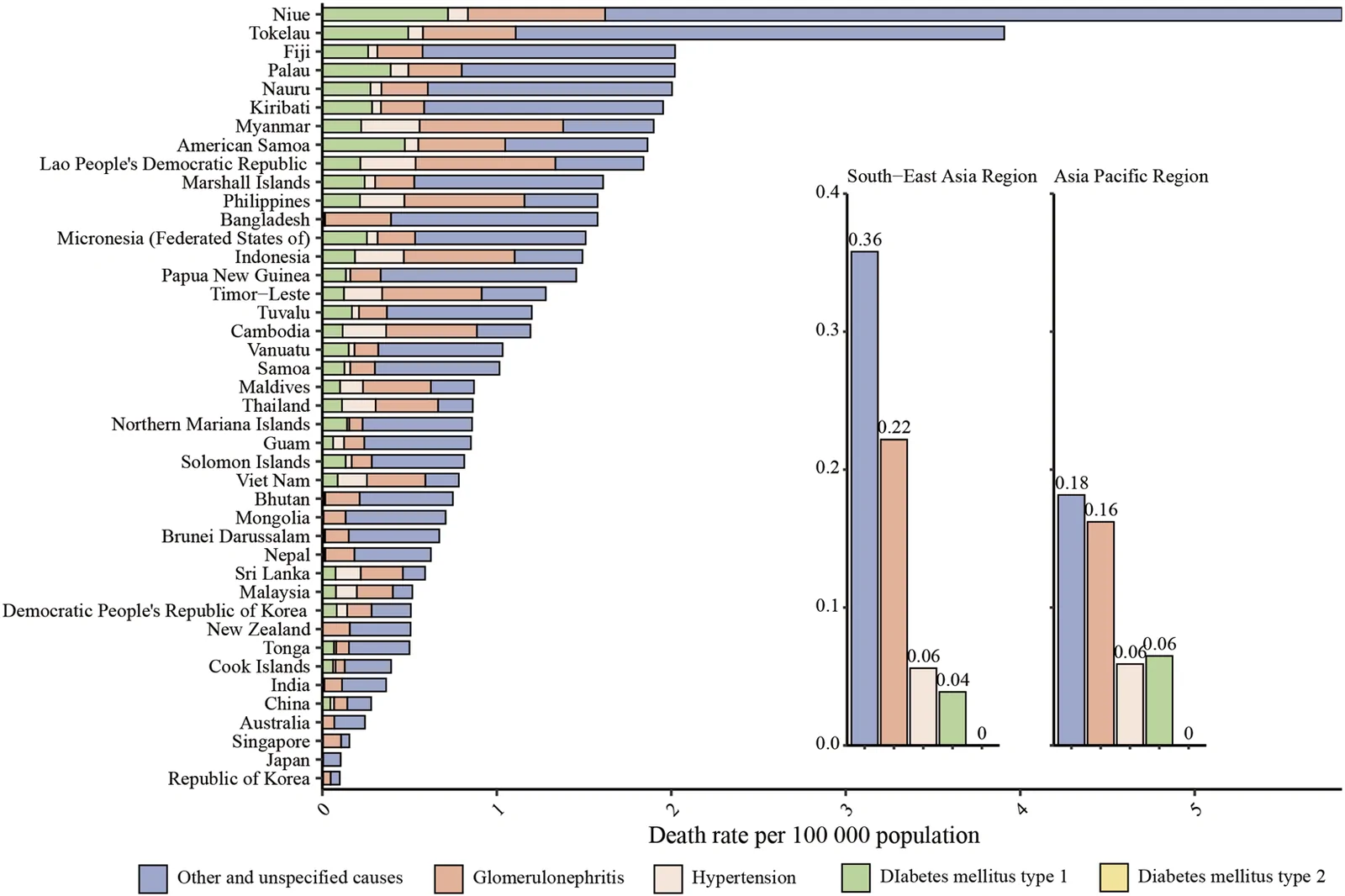
Chronic kidney disease death rate in children and adolescents in Asia-Pacific by country in 2021 (All Asia-Pacific countries and regions. 2021).

### Causal attribution of CKD

CKD of unknown etiology accounted for the largest contribution to the CKD burden in both South-East Asia and the Western Pacific regions (Table 3). The prevalence rates of CKD due to type 1 DM continued to increase in these two regions [AAPC 1.28 (95% CI: 1.19–1.36) in the South-East Asia region and 0.53 (95% CI: 0.43–0.63) in the Western-Pacific region]. In terms of incidence, only CKD due to hypertension showed an increasing trend [AAPC 1.62 (95% CI: 1.52–1.71) in the South-East Asia region and 0.75 (95% CI: 0.28–1.21) in the Western-Pacific region], while the incidence rate of Type 2 DM decreased the most. The death and DALYs rate of CKD for all causes continuously decreased.

**TABLE 3:** The prevalence, incidence, death, and disability-adjusted life-years (DALYs) of chronic kidney disease by cause (South-East Asia region and Western Pacific region. 1990 and 2021).

​	​	South-East Asia Region			Western Pacific Region		
​	​	1990 (95% CI)	2021 (95% CI)	AAPC in rate, 1990 to 2021	1990 (95% CI)	2021 (95% CI)	AAPC in rate1990 to 2021
Prevalence							
​	Type 1 DM	33.96 (45.37–25.15)	46.72 (63.39–33.78)	1.28 (1.19–1.36)	20.25 (27.16–14.78)	23.65 (32.33–16.94)	0.53 (0.43–0.63)
​	Type 2 DM	62.71 (83.61–46.11)	49.43 (68.52–35.45)	−0.99 (−1.16 to −0.81)	43.63 (58.49–31.9)	23.58 (32.93–16.68)	−1.96 (−2.4 to −1.51)
​	Hypertension^a^	5.23 (6.51–4.14)	6.05 (7.87–4.63)	0.45 (0.35–0.54)	4.85 (5.84–3.99)	3.24 (4.01–2.59)	−1.06 (−1.54 to −0.57)
​	Glomerulonephritis^a^	50.05 (57.87–43.29)	49.12 (58.01–41.81)	−0.11 (−0.22 to −0.01)	42.56 (49.44–36.84)	28.81 (33.83–24.86)	−1.32 (−1.45 to −1.18)
​	Other and unspecified causes	633.25 (770.64–521.11)	769.04 (944.68–623.51)	0.67 (0.6–0.74)	448.33 (541.05–372.94)	396.13 (486.98–324.71)	−0.24 (−0.5 to 0.01)
Incidence							
​	Type 1 DM	2.19 (3.26–1.34)	1.92 (2.86–1.2)	−0.41 (−0.49 to −0.34)	1.99 (3.12–1.17)	1.63 (2.49–1.04)	−0.21 (−0.56 to 0.14)
​	Type 2 DM	0.04 (0.07–0.02)	0.04 (0.06–0.02)	−0.53 (−0.77 to −0.29)	0.04 (0.06–0.01)	0.02 (0.03–0.01)	−1.73 (−2.22 to −1.24)
​	Hypertension	0.1 (0.15–0.05)	0.16 (0.24–0.09)	1.62 (1.52–1.71)	0.07 (0.14–0.03)	0.08 (0.13–0.04)	0.75 (0.28–1.21)
​	Glomerulonephritis	7.46 (9.31–5.9)	6.7 (8.3–5.44)	−0.37 (−0.45 to −0.3)	6.8 (8.39–5.21)	5.52 (6.9–4.25)	−0.28 (−0.62 to 0.06)
​	Other and unspecified causes	17.9 (21.87–14.47)	18.13 (21.87–15)	−0.07 (−0.12 to −0.02)	15.44 (18.41–12.49)	13.67 (16.48–11.08)	−0.11 (−0.39 to 0.17)
Death							
​	Type 1 DM	0.06 (0.08–0.04)	0.04 (0.06–0.02)	−1.43 (−1.56 to −1.3)	0.14 (0.2–0.09)	0.06 (0.09–0.04)	−2.57 (−2.74 to −2.4)
​	Type 2 DM	0 (0–0)	0 (0–0)	−0.66 (−1 to −0.32)	0 (0–0)	0 (0–0)	−2.16 (−2.64 to −1.68)
​	Hypertension	0.06 (0.1–0.04)	0.06 (0.09–0.03)	−0.65 (−0.89 to −0.41)	0.08 (0.13–0.04)	0.06 (0.09–0.03)	−0.86 (−1.22 to −0.5)
​	Glomerulonephritis	0.46 (0.61–0.26)	0.22 (0.28–0.15)	−2.33 (−2.44 to −2.22)	0.35 (0.45–0.25)	0.16 (0.21–0.11)	−2.43 (−2.48 to −2.39)
​	Other and unspecified causes	0.92 (1.18–0.47)	0.36 (0.44–0.28)	−3.03 (−3.15 to −2.91)	0.58 (0.7–0.45)	0.18 (0.23–0.14)	−3.85 (−4.02 to −3.69)
DALYs							
​	Type 1 DM	4.86 (6.82–3.04)	3.13 (4.48–1.94)	−1.46 (−1.58 to −1.33)	11.05 (15.36–7.29)	5 (6.97–3.23)	−2.59 (−2.74 to −2.44)
​	Type 2 DM	0.04 (0.06–0.02)	0.02 (0.04–0.01)	−1.72 (−1.97 to −1.47)	0.05 (0.08–0.03)	0.02 (0.03–0.01)	−3.75 (−4.22 to −3.28)
​	Hypertension	5.3 (7.92–3.25)	4.75 (7.05–2.87)	−0.52 (−0.78 to −0.26)	6.49 (10.08–3.77)	4.62 (7.06–2.74)	−0.92 (−1.29 to −0.55)
​	Glomerulonephritis	39.7 (52.64–22.99)	19.39 (24.17–14.15)	−2.33 (−2.45 to −2.2)	29.44 (37.9–21.81)	13.63 (17.42–9.96)	−2.45 (−2.5 to −2.4)
​	Other and unspecified causes	85.75 (108.27–46.04)	36.04 (43.94–28.6)	−2.83 (−2.93 to −2.72)	53.6 (63.75–42.42)	18.57 (22.62–15.03)	−3.53 (−3.67 to −3.4)

### CKD burden by SDI

The 42 countries were grouped into high SDI (six), high-middle SDI (nine), middle SDI (ten), low-middle SDI (thirteen), and low SDI levels (four), with SDI values ranging from 0.41 (Papua New Guinea) to 0.88 (Republic of Korea) (Supplementary Table S1). Among the countries with high SDI, the SDI and CKD burden relationship showed an inverse trend with SDI increasing (Figure 3). However, among the countries with low and low-middle SDI, the CKD incidence rate increased with SDI increasing, while the death and DALYs rates did not show an obvious change. In the middle and high-middle SDI countries, as SDI increased, the CKD incidence rate decreased while the death and DALYs rates increased first and then decreased. In most countries, the prevalence and incidence rates of CKD increased while the death and DALY rates decreased from 1990 to 2021.

**FIGURE 3:**
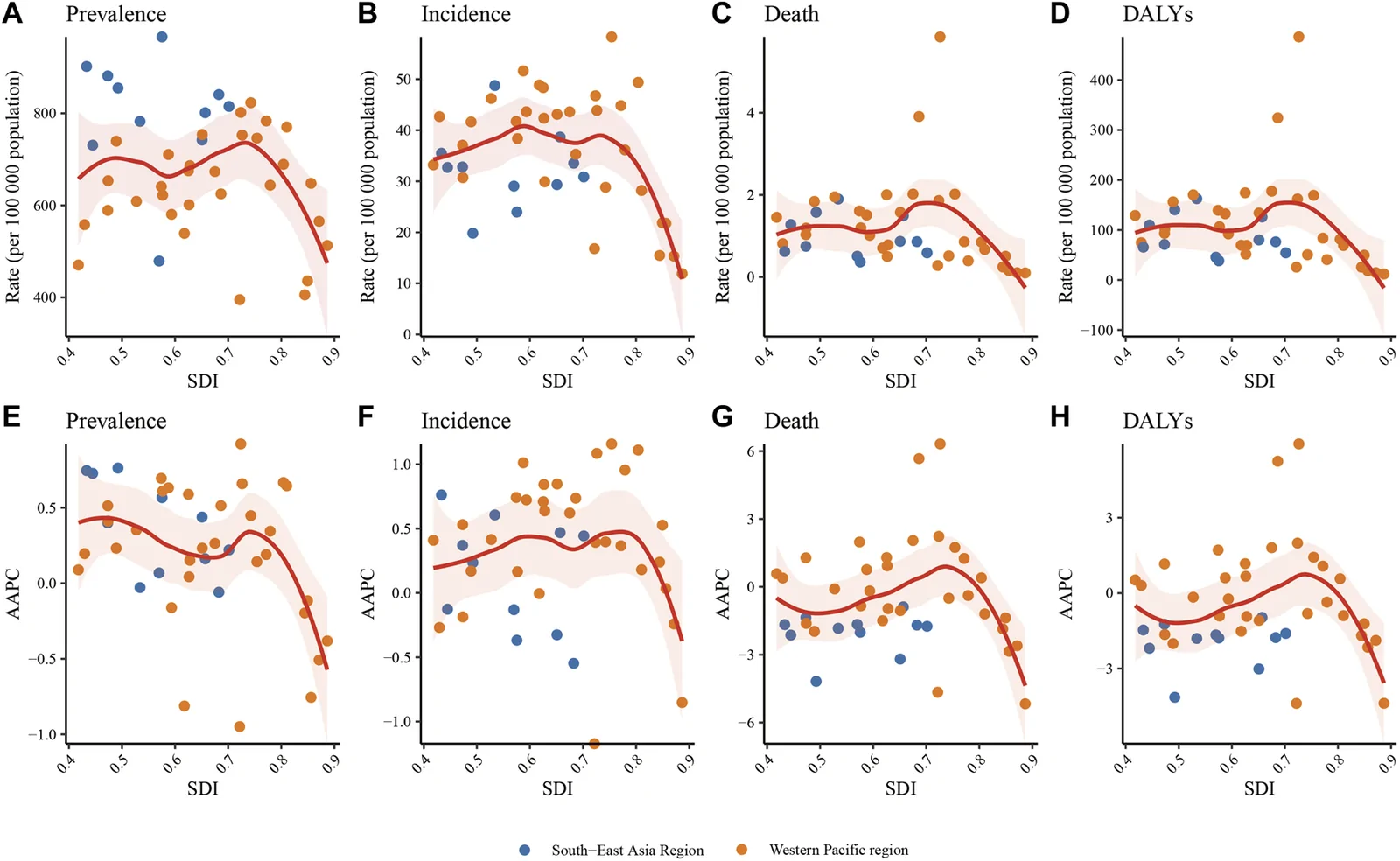
Association between socio-demographic index (SDI) and prevalence **(A)**, incidence **(B)**, death **(C)**, and disability-adjusted life-years (DALYs) **(D)** rate of chronic kidney disease in 2021 and corresponding average annual percentage changes (AAPC) **(E–H)** from 1990 to 2021 (South-East Asia region and Western Pacific region. 1990–2021).

## Discussion

This study systematically analyzed the burden of CKD among children and adolescents younger than 20 years in Asia-Pacific using data from GBD 2021. The overall CKD disease burden decreased from 1990 to 2021, which was inconsistent with the global trend. The previous study found that the global CKD incidence rate among children and adolescents increased, and the Andean Latin America and Central Latin America showed the largest increases [1]. The significant decrease in CKD incidence, death, and DALYs in Asia-Pacific reflected the effectiveness of management measures in this population. Regarding regions, the CKD burden was higher in South-East Asia than in Western Pacific. In terms of age, adolescents aged 15–19 years old had the heaviest CKD burden, and the incidence rate of CKD in this population continued to increase. By SDI quantile, the CKD burden was higher in the middle and high-middle SDI countries.

In recent years, studies on CKD among children and adolescents in the South-East Asia and Western Pacific regions have gradually increased but remain relatively limited overall. Research from the Hong Kong region reports a rising incidence of end-stage kidney disease in children, with prolonged transplant waiting times and mortality risks comparable to other developed countries, particularly elevated among dialysis patients [18]. The spectrum of renal pathology in South Asian children reveals significant regional differences in CKD etiology, suggesting distinct pathophysiological mechanisms [19]. In Sri Lanka, early renal damage in children is closely linked to environmental factors, indicating that chronic kidney disease of unknown etiology may impact pediatric kidney health [20]. Furthermore, the latest Global Kidney Health Atlas highlights the dual pressures of resource scarcity and high demand for kidney replacement therapy in the Asia-Pacific region, underscoring challenges faced by regional health systems [21]. These studies provide essential context for understanding the epidemiology of pediatric CKD in these regions and support our systematic analysis based on the GBD database to address gaps in the literature and inform policymaking.

In 2021, the CKD prevalence rate was 920.36 per 100,000 among the population aged less than 20 years in the South-East Asia region, about 1.94 times higher than in the Western Pacific region (475.41/100,000). The incidence, death, and DALYs rate of CKD was also higher in the South-East Asia region. There were clear socioeconomic differences between the two regions. The South-East Asia region is mainly composed of the countries and territories of middle and lower SDI. In comparison, the Asia Pacific region is mainly composed of the countries and territories of middle and higher SDI. Previous studies suggested that children who grew up in socioeconomically disadvantaged circumstances experienced inequitable burdens, including poorer health, reduced wellbeing, and reduced access to healthcare [22]. The sobering socioeconomic inequalities were discovered in health and renal care for children and adolescents with CKD. For children and adolescents with CKD, the lower socioeconomic position might exacerbate the burden of adverse health and wellbeing outcomes as they were more susceptible to adverse physiological, psychological, behavioral, and other factors [23]. Congenital abnormalities of the kidney and urinary tract (CAKUT) were the leading cause of CKD in children and adolescents. The low SDI countries not only born the highest rate of CAKUT death and DALYs but also were the only SDI group with an increasing absolute number of DALYs [24]. In addition, inequality in access to KRT also existed in these regions, with widespread availability and low out-of-pocket costs in high-income countries and limited access and often high out-of-pocket costs in low- and middle-income countries [21]. In this research, we also found that in the high-SDI countries, the rates of CKD death and DALYs significantly decreased with SDI increasing, consistent with other research. It is essential to reinforce the importance of socioeconomic development for children and adolescents to minimize the adverse influence of socioeconomic inequality. Notably, this study observed a divergent trend in CKD prevalence between South-East Asia and the Western Pacific. In South-East Asia, the increasing prevalence may be partly explained by improved detection and awareness following historically limited screening, as well as improved survival associated with better disease management. In contrast, the decreasing trend in the Western Pacific may be related to more established healthcare systems and more effective prevention and management of CKD and its risk factors [25]. However, these interpretations should be approached with caution, as differences in data quality and reporting practices may also influence the observed trends.

Pacific island countries had a relatively higher CKD incidence and death rate among all countries/regions, even though more than half of them belonged to middle and higher SDI countries. As island countries, the Pacific Island countries were more ecologically fragile and heavily dependent on marine ecology. They were more affected by climate change than mainland countries [25]. Extreme weather caused by climate change posed serious threats to the ecological environment of island countries and increased the disease burden [26]. Growing epidemiological evidence showed that climate change, especially ambient temperature, was associated with the global disease burden. There was a consistent positive correlation between high-temperature exposure and an increased risk of kidney-related morbidity and mortality. For example, high temperature was associated with an increased risk of CKD hospitalization [27, 28], and CKD-related emergency room visits increased in hot weather [28, 29]. One possible explanation was that increased temperatures increased the risk of repeated exposure to high temperatures, leading to dehydration and potential damage to renal function [30]. In addition, environmental pollution and the medical system might also be potential factors leading to this difference. However, the current research on the disease burden in Pacific countries is relatively limited, and further research is needed to explore the factors that influence the CKD disease burden in Pacific Island countries.

The adolescents aged 15–19 years had the heaviest CKD burden in Asia-Pacific, consistent with other studies [1]. In the GBD database, there were two more causes of CKD in this population than in other age groups: type 2 DM and hypertension, which could contribute to the increase in CKD burden. DM and hypertension were the two primary risk factors for CKD [31] and contributed to the deterioration of renal function through various mechanisms such as hyperfiltration, inflammation, and oxidative stress [32]. In addition, the risk of exposure to environmental toxins and pollutants increased with ageing, which was associated with a higher risk of incident CKD in children and adolescents [33]. Routine kidney disease screening in children and adolescents with risk factors for CKD might help the identification and early intervention to prevent or delay its progression.

Children less than 5 years old faced the highest incidence rate of CKD, and the death and DALYs rates in this population were also relatively higher. Common causes of CKD in infancy include congenital renal dysgenesis and obstructive urinary tract abnormalities. A study found that the median age at diagnosis for patients with CAKUT and inherited kidney disease was 1 and 2 years, respectively. More than half of CKD cases were caused by CAKUT [34]. However, the implementation of some therapy strategies for infants faced huge challenges due to their small size, high risk of infection, and multiple complications [35, 36]. Additionally, some other factors could also influence the CKD diagnosis. For example, it was common that GFR was lower in infancy and would be normalized as the child grew. This sometimes might lead to misdiagnosis. Due to the limited ability to communicate symptoms, diagnosing CKD in very young children relied more on laboratory tests and clinical assessments. This could lead to variations in diagnosis practices, including the potential for overdiagnosis [1]. Therefore, it is necessary to conduct a more detailed analysis of the epidemiological characteristics of CKD in children aged 0–4 years, especially infants under 1 year old.

Gender disparities existed in the CKD burden. The death and DALYs rates of CKD were higher in males than females in the Asia-Pacific region, consistent with the global trend. However, the females had higher rates of prevalence and incidence rates in the Western Pacific region, while the Southeast Asia was the opposite. The reasons for gender disparities in disease epidemiological characteristics are complicated, including physiological, socioeconomic, cultural, and other factors. The prevalence of CKD was higher in females worldwide [37–39], while the majority of persons initiating KRT were males, including dialysis or kidney transplants [39]. Most studies suggested that males experienced faster kidney function decline [40, 41], which could result in a higher proportion of advanced stages of CKD [42] in males and increase the clinical application of KRT. In addition, a higher risk of mortality from CKD was also found in males than in females [40]. However, the risk association between all-cause mortality and eGFR decline was stronger in females than in males [43], indicating that females were more likely to die from other CKD-related diseases before reaching the advanced stage of CKD [44]. In addition to biological factors, social, economic, and cultural factors affected the gender disparities of CKD. The socioeconomic factors, as discussed previously, that CKD patients in lower-SDI-level countries faced poor therapy quality and higher costs, which increased the proportion of patients with advanced stage of CKD and further widened the gap between males and females. This study found that the rate of death and DALYs was higher in males than females, which might be related to the faster progression of CKD in males, but this gender gap decreased with the increase of SDI. It should be noted that much of the existing evidence on gender disparities in CKD is derived from adult populations. Given the biological and epidemiological differences between children and adults, the extrapolation of these findings to pediatric populations should be approached with caution. Therefore, the observed gender differences in this study require further validation through pediatric-specific research.

There are also many limitations in this research. First, the causes of CKD in GBD do not fully reflect the epidemiology of CKD in children. Other common causes of CKD, such as CAKUT and hereditary kidney diseases, have not been distinguished in detail. Especially for infants under 1 year old, congenital factors have a great influence. GBD 2021 also lacks data on CKD caused by type 2 diabetes mellitus and hypertension among the population under 15 years old. This limitation may reduce the accuracy of etiological analyses in younger populations and restrict our ability to fully characterize cause-specific patterns of CKD in children, particularly those under 15 years of age. Therefore, the interpretation of etiological findings in this age group should be approached with caution. In addition, a substantial proportion of CKD cases were categorized as “other and unspecified causes,” which further constrains the interpretation of etiological patterns. This issue is likely related to limitations in data availability, diagnostic capacity, and cause attribution, particularly in resource-limited settings. The dominance of this category may limit the usefulness of the findings for developing targeted, cause-specific public health interventions, and thus the etiological results should be interpreted with caution. Second, the epidemiological data in the GBD 2021 database are based on speculation based on mathematical models, which deviate from the actual situation to a certain extent. Although the GBD study uses standardized modeling approaches to integrate multiple data sources, these estimates are subject to uncertainty and potential bias, particularly in regions with limited or low-quality primary data. This may affect the robustness and comparability of the results across different settings. Therefore, our findings should be interpreted with caution, especially in low-SDI regions. Third, GBD only identifies CKD based on a single eGFR/ACR, which may lead to partial misclassification. The use of standardized eGFR and ACR thresholds derived from GBD estimates, combined with variations in laboratory methods, measurement techniques, and calibration standards across regions, may introduce additional measurement bias and misclassification, which may cause the data to have a certain gap from the actual situation. In addition, the interpretation of gender disparities in this study may be limited by the lack of pediatric-specific evidence. Some explanations are informed by findings from adult populations, and given the biological and epidemiological differences between children and adults, such extrapolation should be approached with caution. Therefore, the observed gender differences require further validation in pediatric-specific studies. Finally, the availability and quality of primary epidemiological data used in the GBD vary considerably across countries. In locations where empirical data are sparse or absent, GBD relies heavily on out-of-sample predictive modeling, which may introduce greater uncertainty and potential bias in estimates. Although statistical methods attempt to minimize instability, variations in reporting accuracy, measurement methods, and case definitions can lead to deviations from actual burden, particularly in data-poor settings. These limitations should be taken into account when interpreting the findings.

In summary, we comprehensively evaluated the epidemiological characteristics of CKD in children and adolescents across the Asia-Pacific region from 1990 to 2021. While the overall CKD burden declined, substantial disparities persist. Pacific island countries exhibited a persistently high burden, and low-SDI countries showed the least improvement. Males consistently experienced higher mortality than females, and adolescents aged 15–19 years bore a heavier burden than younger children. These findings underscore the need for age- and gender-specific interventions, with particular attention to low-SDI and Pacific regions, to reduce preventable deaths and improve long-term outcomes in young CKD patients. Targeted public health strategies, including improved early screening and enhanced access to pediatric kidney care in high-burden regions such as Pacific Island countries, may help reduce these disparities. Strengthening primary healthcare capacity, promoting early-life risk factor management, and improving access to timely referral and treatment services may further support effective CKD prevention and control in children and adolescents [45–47].
